# Our Eye is on the Future

**DOI:** 10.4103/0974-9233.61208

**Published:** 2010

**Authors:** Deepak P. Edward

**Affiliations:** Editor-in-Chief, MEAJO and Professor of Ophthalmology, Northeastern Ohio University College of Medicine and Pharmacy, Department Chair of Ophthalmology, Summa Health Systems, Akron, OH USA

It is that time of the year when we reflect on the past with an eye to the future. The past has been short and it seems like yesterday when the inaugural issue of the Middle East African Journal of Ophthalmology (MEAJO) was published online, and thereafter the print edition made its debut at the MEACO meeting in Bahrain. A group of highly enthusiastic and motivated members of the editorial board and International Advisory Board attended the meeting and resolved that they would work towards making MEAJO the flagship journal of MEACO. This prescient group of individuals had a vision to publish high-quality, peer reviewed, evidence-based manuscripts focused on research related to issues in the region, and also publish theme-based reviews that would educate the ophthalmology community in the Middle East and Africa. With the help of the editorial and advisory board, the editorial team, the global community of scientific reviewers and most importantly the authors who submitted their work for publication, we have made great strides in creating a Journal of Excellence in the last year.

This is reflected by the privilege of now being listed on PubMed with which comes great responsibility to ensure that the culture of excellence is maintained.

In 2009, 169 manuscripts were submitted to MEAJO online which represents more than a threefold increase in submissions compared to the previous year. Manuscripts were submitted by authors from the region and internationally including Bahrain, Egypt, Iran, India, Japan, Jordan, Kenya, Kuwait, Morocco, Nigeria, Oman, Turkey, Tunisia, Saudi Arabia, United States, and Yemen. The scope of submission indicates the broad reach of the Journal facilitated by online submission and access. In keeping the highest standards of peer review, all submissions continue to be reviewed in a double blinded manner. The acceptance rate for the Journal was 33% in 2009. In the second quarter of 2009, we published the first theme-based edition-a review of glaucoma surgery-followed by a comprehensive update on uveitis contributed by the Society for Ophthalmo-Immunoinfectiology in Europe. The current issue features an update on corneal disease and refractive surgery review articles from well-known specialists across the globe. Future issues in 2010 will address pediatric ophthalmology and plastics, retina, ocular oncology and public health. Such theme-based issues allow the ophthalmic community to access current treatment and surgical paradigms in a timely manner that may otherwise take years to publish in a textbook. Our hope is that these theme-based issues prove to be an excellent clinical reference occupying space among the trusted manuals on the surgeon's book shelf.

We also gauge the effectiveness of a new journal by other metrics provided by the publisher. The average time from submission to acceptance was 73 days in 2009. The Journal in the past year has been accessed 6000-8000 times per month. The number of individuals requesting the table of contents continues to increase monthly. This online activity portends the growing importance of a fledgling Journal such as MEAJO. More importantly such activity confirms the requirement of such a Journal for the region. It is not a question of if the Journal was required but rather how can it best educate and enhance clinical and surgical practice within the Middle East and Africa.

In consideration of the economic issues that often face countries in this region and the high priority the Council places on providing educational opportunities to its membership, it was resolved that the Journal remain an open access online Journal.^1^ Furthermore, the print version is published on high-quality eco-friendly paper and made available without charge to the various societies in the region.

One of the challenges identified in the past year has been working with authors in regions where English is not the native language. Although the science behind the submission is often excellent and warrants peer review, the language often required professional editing. MEAJO, with the assistance of the Executive board, now provides an English language editing service for articles that have scientific merit. The editing service has already helped several contributors refine their submissions. The Journal continues to emphasize the importance of study approval by institutional review boards, ethic committees, or animal care committees where appropriate. Although regional differences in the interpretation and recommendations exist, the documentation of such a review remains mandatory and a high priority for manuscripts received by the Journal.^2^ At the beginning of this New Year, I would like to personally thank the members of the editorial and advisory board and the scientific reviewers for contributing their time and effort in this educational journey.
